# Supplementary figures and images for: A Randomized, Single-Blind, Group Sequential, Active-Controlled Study to Evaluate the Clinical Efficacy and Safety of α-Lipoic Acid for Critically Ill Patients With Coronavirus Disease 2019 (COVID-19)
Source: Front Med (Lausanne). 2022 Feb 4;8:566609. doi: 10.3389/fmed.2021.566609 (PMC8854372; doi:10.3389/fmed.2021.566609)

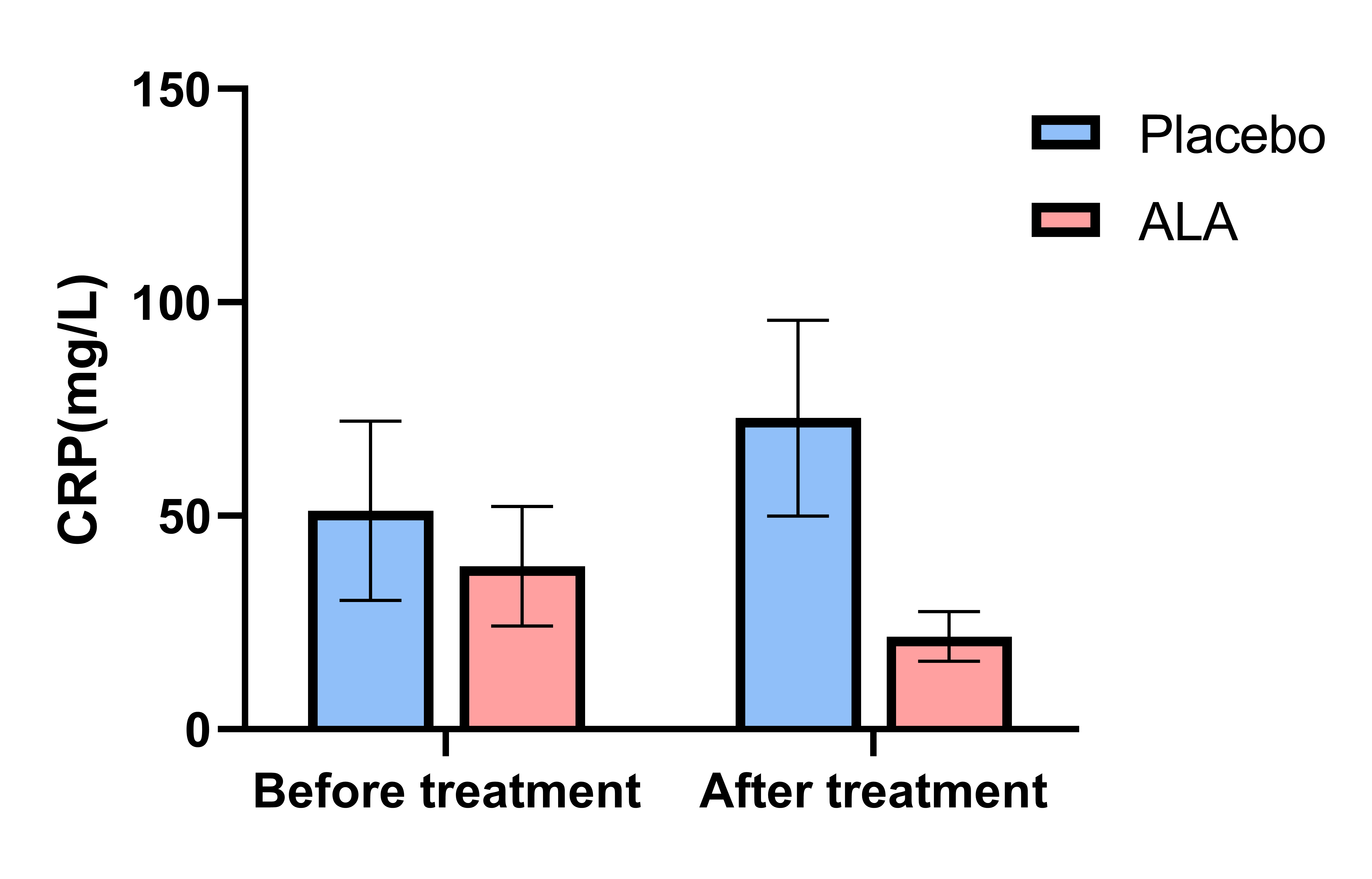

Supplement: Supplementary Figure 1 — CRP. [file Image_1.TIF]

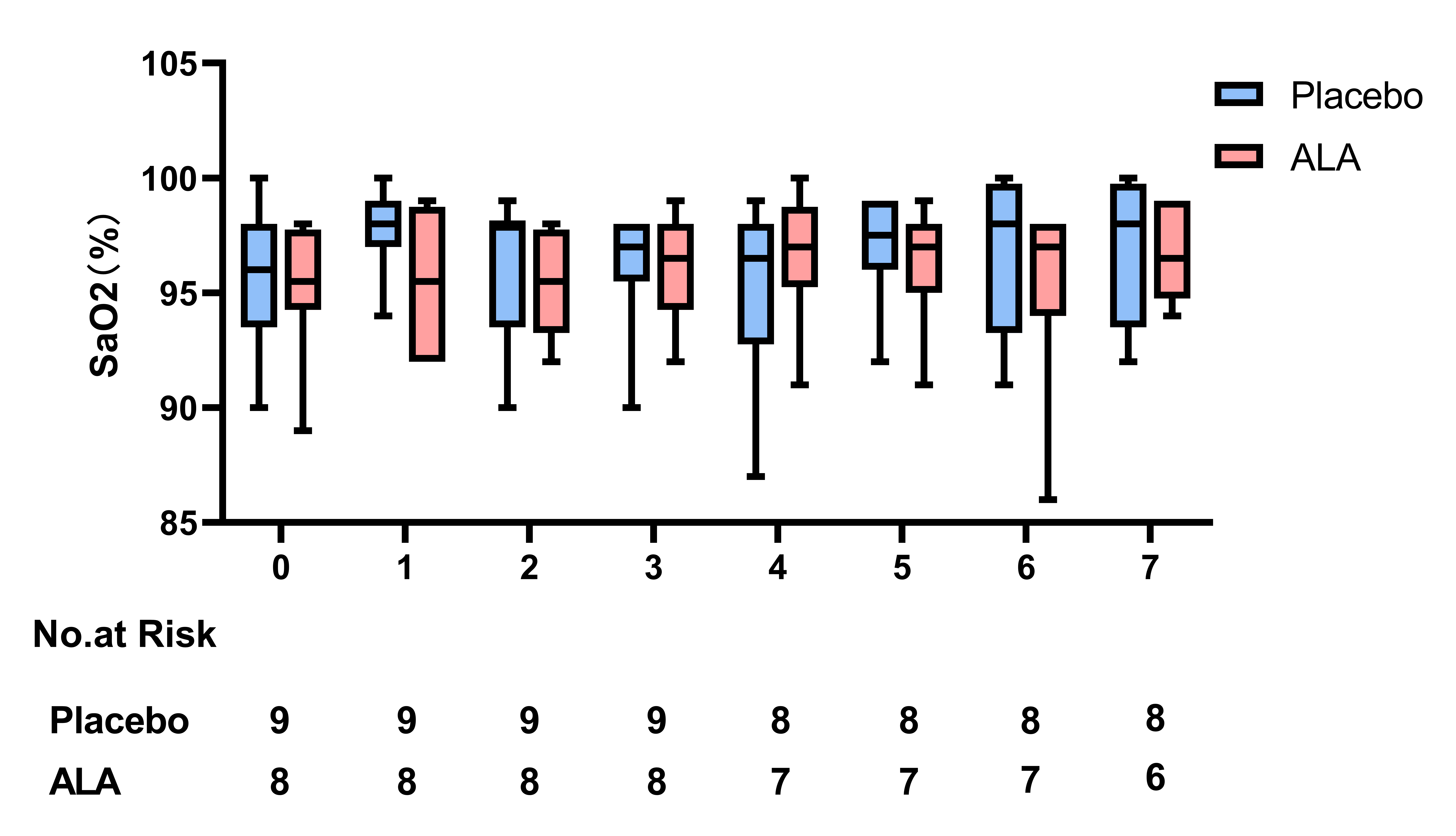

Supplement: Supplementary Figure 2 — SaO2. [file Image_2.TIF]
